# Genome-wide analysis of the response to ivermectin treatment by a Swedish field population of *Haemonchus contortus*

**DOI:** 10.1016/j.ijpddr.2021.12.002

**Published:** 2021-12-23

**Authors:** Paulius Baltrušis, Stephen R. Doyle, Peter Halvarsson, Johan Höglund

**Affiliations:** a Department of Biomedical Sciences and Veterinary Public Health, Section for Parasitology, Swedish University of Agricultural Sciences, P.O. Box 7036, Uppsala, Sweden; b Wellcome Sanger Institute, Hinxton, Cambridgeshire, CB10 1SA, UK

**Keywords:** *Haemonchus contortus*, Ivermectin, Whole-genome sequencing, Anthelmintic resistance, Pool-seq

## Abstract

*Haemonchus contortus* is a pathogenic gastrointestinal nematode of small ruminants and, in part due to its capacity to develop resistance to drugs, contributes to significant losses in the animal production sector worldwide. Despite decades of research, comparatively little is known about the specific mechanism(s) driving resistance to drugs such as ivermectin in this species. Here we describe a genome-wide approach to detect evidence of selection by ivermectin treatment in a field population of *H. contortus* from Sweden, using parasites sampled from the same animals before and seven days after ivermectin exposure followed by whole-genome sequencing. Despite an 89% reduction in parasites recovered after treatment measured by the fecal egg count reduction test, the surviving population was highly genetically similar to the population before treatment, suggesting that resistance has likely evolved over time and that resistance alleles are present on diverse haplotypes. Pairwise gene and SNP frequency comparisons indicated the highest degree of differentiation was found at the terminal end of chromosome 4, whereas the most striking difference in nucleotide diversity was observed in a region on chromosome 5 previously reported to harbor a major quantitative trait locus involved in ivermectin resistance. These data provide novel insight into the genome-wide effect of ivermectin selection in a field population as well as confirm the importance of the previously established quantitative trait locus in the development of resistance to ivermectin.

## Highlights

• Pre- and post-treatment populations were genetically similar despite 89% reduction.
• Pairwise gene and SNP frequency differentiation identified in chr4.
• Difference in nucleotide diversity found in ivermectin QTL region in chr5.

## Graphical abstract

**Figure:**
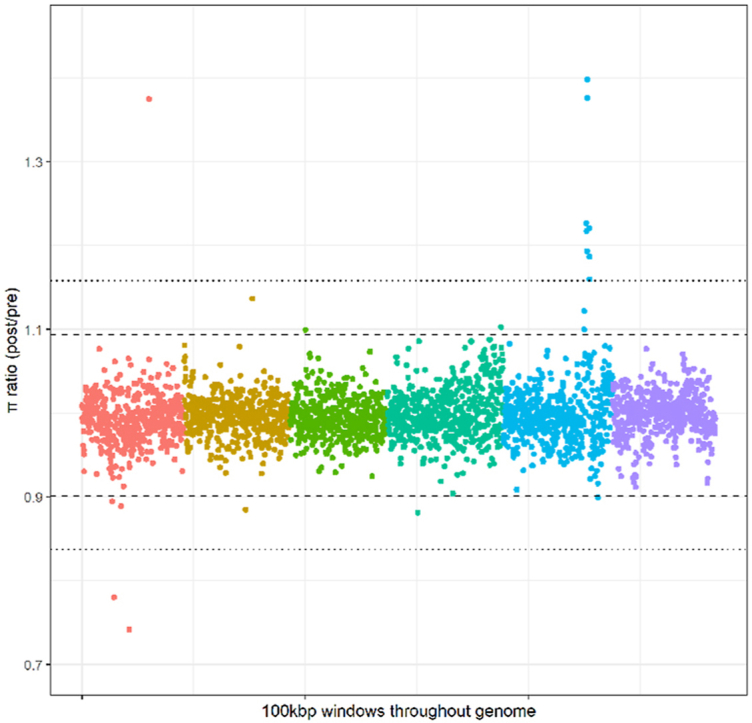

## 1 Introduction

The nematode *Haemonchus contortus* is one of the most pathogenic helminth species of small ruminants across the world. Together with other commonly found gastrointestinal nematodes (GINs), *H. contortus* contributes to reduced weight gain in infected individuals, the consequences of which often result in the decreased production of meat and dairy products. Severe cases of infection with *H. contortus* lead to anemia and can even result in sudden death of the host. Infection by GINs has a staggering impact on animal husbandry, estimated at approximately €686 million in treatment and prevention costs annually in Europe alone (Charlier et al., 2020). *H. contortus* is a cosmopolitan GIN, partly due to the species capacity to adapt to both colder and warmer climates, to infect a number of different host species, and to rapidly develop resistance to every drug class currently used to control it (Van den Brom et al., 2013; Geurden et al., 2014; Cazajous et al., 2018; Höglund et al., 2020).

Despite the broad impact on animal health and production, our understanding of the mechanisms by which resistance evolves, especially to certain drugs such as ivermectin (IVM), is generally lacking. Although numerous candidate genes have been proposed over the years as putative drivers of resistance to IVM in *H. contortus* (summarized in Doyle et al., 2019), the evidence for the involvement in IVM resistance for some of these candidate genes remains inconclusive. For example, some genes initially proposed to be associated with resistance, for example, *dyf-7, lgc-37, glc-5, avr-14,* have subsequently been shown not to be significantly associated with the resistant phenotype in follow-up studies (Laing et al., 2016; Rezansoff et al., 2016; Elmahalawy et al., 2018). The many genes proposed to be associated with resistance has led to a widely accepted view that IVM resistance may be caused by one of a number of changes throughout the genome, including genetic changes in both coding and non-coding sequences. The high genetic diversity among GINs such as *H. contortus*, together with the fact that many studies focus on only a few individuals among limited, yet genetically and phenotypically diverse strains, suggests that some candidates may be falsely associated with resistance. Similarly, approaches to identify genetic markers associated with resistance can also produce false associations; even if a genetic marker is linked to a causal variant, a lack of understanding of linkage disequilibrium (LD) patterns among variation in genomes of individuals in the studied populations (Doyle and Cotton, 2019) and the potential changes in these patterns over time and in different populations, may result in differences in the degree of linkage, and in some cases, the associated variants becoming unlinked from the causal variants. Candidate gene approach studies which focus only on the frequency changes of single target genes and/or mutations (Blackhall et al., 1998; Eng et al., 2006; Urdaneta-Marquez et al., 2014), without taking into account the genome-wide genetic variation, are limited. Thus, the current gaps in the understanding of the mechanism of IVM resistance cannot simply be addressed through conventional candidate gene approaches alone.

Genome-wide studies of genetic variation have begun to provide a more comprehensive and unbiased framework towards identifying genomic regions associated with quantitative, phenotypic traits of interest (i.e. Quantitative trait loci, QTL), including genes associated with anthelmintic resistance in veterinary nematodes. These advances, made possible by technological improvements and rapid decrease in the cost of sequencing, have already provided important insight in the genetics of trait variation in other fields, including the study of important human pathogens (Chevalier et al., 2014; Abkallo et al., 2017; Anderson et al., 2018). Several studies, performed on other parasitic worm species (Choi et al., 2017; Doyle et al., 2017) as well as protozoans (Cheeseman et al., 2015), have similarly shown the capacity of genome-wide approaches in combination with relevant statistical analyses to identify genomic regions under selection as well as propose genes associated with drug resistance. For *H. contortus*, the availability of the high-quality reference genome (Doyle et al., 2020) and genetic tractability via the ability to perform genetic crosses (Redman et al., 2012), has accelerated research to refine the large list of candidate genes to identify specific discrete regions and, in some cases, individual genes and alleles responsible for driving resistance to several anthelmintics. For example, genetic crossing together with whole-genome sequencing has been used to map monepantel resistance (Niciura et al., 2019), which identified a single major locus in chromosome 2 (7.2–8.7 Mbp) containing the previously reported *mnptl-1, deg-3* and *des-2* genes thought to be involved in monepantel resistance development (Kaminsky et al., 2008; Rufener et al., 2009). For IVM, a backcross experiment followed by sequencing identified a single QTL in chromosome 5 (37–42 Mbp) associated with a resistance phenotype in two geographically distinct *H. contortus* strains (Doyle et al., 2019) which has since been refined to approximately 300 kbp with a putative driver identified among 24 genes within the QTL (Doyle et al., 2021). One concern of the use of the genetic crosses is that evidence of selection (i.e. the identified QTL, linked to the resistance phenotype) may not be representative of selection acting on the parasites in the field setting, and thus evidence outside of the laboratory is necessary. Although the chromosome 5 QTL has been replicated in field populations in the US (Doyle et al., 2021) and other field populations phenotypically defined for IVM resistance (Sallé et al., 2019), a study of *H. contortus* from China has identified multiple genomic regions in each chromosome between the phenotypically resistant and susceptible to IVM field isolates (Khan et al., 2020). Even though genome-wide scans suffer from many of the same complications as candidate gene studies (Doyle and Cotton, 2019), i.e. direct comparison of resistant and susceptible strains will identify many regions of the genome which differ due to their unique evolutionary histories and not due to resistance (Doyle et al., 2019), field validation of QTL identified in genetic crosses will strengthen the association between genetic variants and resistance. Together, these data will provide greater confidence in prioritizing variants for the development of molecular diagnostics used to monitor the evolution and spread of resistant alleles in the field (Kotze et al., 2020).

Motivated by the recent progress made in QTL mapping using genetic crosses and need for further validation, here we describe an approach to detect and characterize IVM-mediated selection in a field population of *H. contortus* from Sweden. By performing pooled whole-genome sequencing on infective stage larvae of the same population pre- and post-IVM treatment, we measured changes in nucleotide diversity and allele frequencies in response to treatment throughout the genome.

## 2 Materials and methods

### 2.1 Sample collection and parasitological analysis of phenotypic resistance using the FECRT

A commercial sheep farm in the south-eastern part of Sweden was chosen for this study, due to the presence of suspected but unconfirmed IVM treatment failure as well as high egg counts both pre- and post-treatment of which ≥90% belonged to *H. contortus*. Macrocyclic lactones (which included ivermectin) had been used extensively since at least 2012 to treat GIN infections in animals.

Field *H. contortus* samples were obtained pre-treatment and seven days post-treatment with IVM (200 μg/kg) from the same flock of sheep (n = 11) in connection with fecal egg count reduction testing (FECRT). Oral drenching of the sheep and the collection of fecal samples was carried out by the animal owner under the supervision of a veterinarian. Fecal samples were sealed in plastic bags and sent to a local diagnostic laboratory (*Vidilab* AB) where they were processed to purify the eggs, after which egg counts were performed using a modified McMaster method in accordance with Ljungström et al. (2018). Fecal egg count reduction from pre-to post-treatment was then calculated using the R package *eggCounts* (v. 2.3.).

The remaining fecal material not used in the FECRT was pooled into either pre- or post-treatment categories and cultured to facilitate egg hatching, after which the infective third stage larvae (L3) were harvested as previously described (Halvarsson and Höglund, 2021).

### 2.2 DNA extraction, sequencing library preparation, and whole-genome sequencing

The pre- and post-treatment pools of L3 were each subdivided into four microcentrifuge tubes, containing approximately the same packed volume representing thousands of individual larvae (>1000) per tube. This generated eight samples in total, consisting of four technical replicates for each of the pre-treatment and post-treatment categories. Genomic DNA was extracted from the eight samples using the Nucleospin Tissue kit (Macherey-Nagel), following the guidelines issued by the manufacturer. The DNA concentration for each replicate for the pre-treatment category was - 2.3 ng/μl, 9.46 ng/μl, 2.64 ng/μl and 2.78 ng/μl; whereas for the post-treatment – 53 ng/μl, 12,1 ng/μl, 65 ng/μl and 17.9 ng/μl.

The genomic DNA samples for each replicate were sent to *Annoroad Gene Technology* (AGT) (Beijing, China) for sequencing. The sequencing library was prepared using the Illumina DNA Prep kit and was sequenced using one lane of an Illumina NovaSeq 6000 using 150 bp paired-end sequencing chemistry. Sequencing yielded 231.9 Gbp of data in 1.546 billion reads. Prior to data delivery, AGT prefiltered the data to remove adapter sequences, low-quality reads where more than 50% of the bases had a Q ≤ 19 and reads containing more than 5% missing bases (N).

### 2.3 Sequencing data analysis

The FASTQ files for the eight samples were mapped onto the *H. contortus* reference genome (available at: https://parasite.wormbase.org/Haemonchus_contortus_prjeb506/Info/Index/; Doyle et al., 2020) using *bwa-mem (v.0.7.17)* (Li and Durbin, 2009). *Picard (v. 2.23.4;* https://github.com/broadinstitute/picard) was used to remove duplicate reads and only perfectly mapped reads pairs (i.e. reads that are properly aligned in a pair, within 1000 bp of one another) were retained for further analysis (*samtools* (v.1.10; http://www.htslib.org/) *view -f 2*). Comparisons within and between treatment groups involving the mitochondrial genome were not considered.

For subsequent nucleotide diversity (ℼ), Tajima's D statistic, pairwise genetic differentiation comparisons (F_ST_ and Fisher's exact test) analyses, the BAM files from the four replicates per each treatment category were merged into a single pre- or post-treatment group using *samtools merge*. For the CMH test, the replicates were kept separate (as described below).

Pileup file generation for the sequence data analysis with either *NPstat* (Ferretti et al., 2013) or *Popoolation2* (Kofler et al., 2011) tools has been described previously (Doyle et al., 2019). In short, pileup files were generated using *samtools mpileup* (*-d 500 --min-MQ 30 --min-BQ 30 --adjust-MQ 50*).

### 2.4 Analyses of within group genetic diversity and between group genetic differentiation

Within group genetic diversity was determined using *NPstat (v.1),* from which nucleotide diversities as well as Tajima's D statistic within the two treatment groups was determined in 100 kb windows throughout the genome (*-n 200 -l 100000 -maxcov 500 -minqual 20*). This analysis was performed per chromosome per sample by splitting the mpileup and running the analysis on each respective part of the data separately, before merging the output.

Pairwise genetic differentiation between the pre-treatment and post-treatment sample groups was determined using *Popoolation2*. The previously generated pileup files were converted into synchronized files (*popoolation2 mpileup2sync.pl --min-qual 20*) and sequence around indels +5 bp (*popoolation2 identify-indel-regions.pl --min-count 2 --indel-window 5)* was removed (*popoolation2 filter-sync-by-gtf.pl*). The synchronized file containing the data for each of the eight samples was used to perform the Cochran-Mantel-Haenszel (CMH) test in order to investigate for the presence of any consistent allele frequency changes between the replicates belonging to each of the two treatment groups (*cmh-test.pl --min-count 4 --min-coverage 20 --max-coverage 2%*). However, since the replicates were previously pooled, they do not represent true, distinct pairs (from the same animals) and were, therefore, compared in an arbitrary fashion. In a similar way (but using a synchronized file containing the merged replicates), per nucleotide allele frequency differences between the two treatment groups were estimated using Fisher's exact test (FET) (*fisher-test.pl --min-count 4 --min-coverage 20 --max-coverage 2% --suppress-noninformative*). Pairwise F_ST_ values were also determined throughout the genome in 10,000 bp windows using a step size of 5000 bp (*popoolation2 fst-sliding.pl --pool-size 1000 --min-count 4 --min-coverage 20 --max-coverage 2% --window-size 10000 --step-size 5000*) and per genes (*popoolation2 fst-sliding.pl --pool-size 1000 --min-count 4 --min-coverage 20 --max-coverage 2% --window-size 1000000 --step-size 1000000*) after having created a synchronized file using *H. contortus* genome annotation (available at: https://parasite.wormbase.org/Haemonchus_contortus_prjeb506/Info/Index/; WBP15) as an input (*popoolation2 create-genewise-sync.pl*).

SNP calling was performed separately using *bcftools* (v. 1.12; http://www.htslib.org/doc/bcftools.html; *bcftools mpileup -d 500 --min-MQ 30 --min-BQ 30 --adjust-MQ 50 -a FORMAT/DP | bcftools call -mv | bcftools view -i '%QUAL*>=*20 & FORMAT/DP*>=*20′*) and the called SNPs were annotated using SNPEff (Cingolani et al., 2012) (v.4.3t; http://pcingola.github.io/SnpEff/).

### 2.5 Data and statistical analysis

Data was visualized using the R package ggplot2 (v. 3.3.3; https://github.com/tidyverse/ggplot2) in Rstudio (v. 1.2.5033). Nucleotide diversity and Tajima's D ratios as well as F_ST_ measurements per genomic window were determined to be substantially different if higher than genome-wide (or gene-wide) mean + 3 standard deviations (SD) and/or genome-wide (or gene-wide) mean + 5 SD values. Significance (p ≤ 0.05) in the case of FET and CMH test was determined using a genome-wide Bonferroni's correction ($=\frac{0.05}{number\;of\;genome-wide\;SNPs}$ ).

Full code used in the different steps of the analyses together with explanations is available at https://github.com/pauliusbaltrusis/WGanalysis.

## 3 Results

### 3.1 Measurement of resistance by FECRT and whole-genome sequencing of pre- and post-treatment populations

We first set out to determine the efficacy of IVM on the treated farm by performing a FECRT. This was important to confirm the presence of resistance on this farm for which resistance was suspected. The average egg counts per gram of feces were 21568 ± 16313 before treatment and 2327 ± 3094 after treatment, resulting in an egg count reduction of 89% (approximate 95% confidence interval = 71.7–95.8%), consistent with the presence of resistance (Coles et al., 1992).

We sequenced pools of larvae collected pre- and post- IVM treatment, represented by four technical replicates at each time point for a total of eight pools. In total, we obtained 1.5 × 10^9^ raw reads which on average 89.5% mapped to the genome; after processing and deduplication, an approximate 52.5 × coverage per chromosome (excluding mtDNA) per replicate pool was achieved. Analysis of genetic variation within the pooled mapped sequencing data identified up to approximately 2.7 million (in the case of FET) biallelic SNPs across all eight replicates.

### 3.2 Genome-wide genetic diversity within the pre- and post-treatment populations

**Fig. 1:**
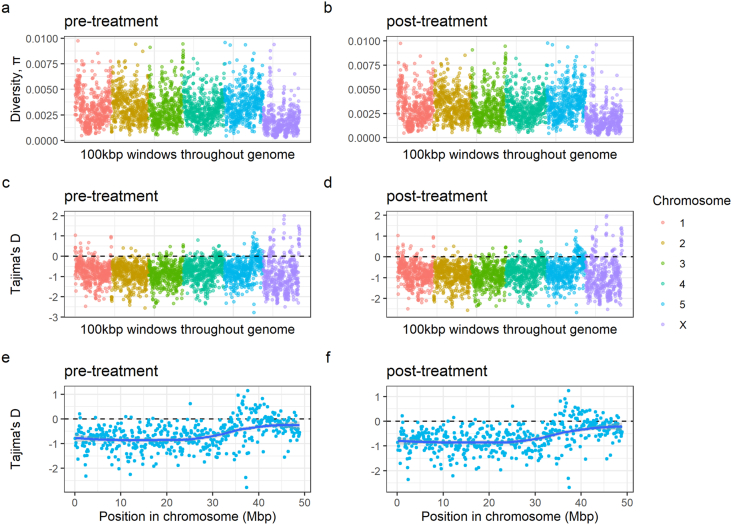
**Genetic diversity within pre- and post-treatment groups.** Within treatment group nucleotide diversity (a and b) and Tajima's D (c and d) comparisons per 100 kbp genomic windows per chromosome based on pooled sequencing of *H. contortus* L3, recovered before and seven days after IVM treatment from the same flock of sheep. (e and f) Tajima's D estimates per 100 kbp genomic window were evaluated specifically for chromosome 5. The blue line represents a LOESS function drawn through the data points. The dashed line in (c and d) and (e and f) is drawn through value 0 which indicates neutrality and otherwise serves to separate windows wherein Tajima's D is either positive or negative. (For interpretation of the references to colour in this figure legend, the reader is referred to the Web version of this article.)

To understand the effect of treatment on genetic diversity within each group, we calculated nucleotide diversity (ℼ) as well as Tajima's D for each group (Fig. 1a and b and Fig. 1c and d). No significant differences between the ℼ value distributions for all chromosomes were observed between both treatment groups (*p*-value = 1; one-sample Kolmogorov-Smirnov test). We did note that the average diversities for the X chromosome were approximately half (ℼ = 0.001 ± 0.001 for both pre- and post-treatment) of those observed for the autosomes (for both pre- and post-treatment ℼ = 0.003 ± 0.001), consistent with a previous study (Doyle et al., 2020). Mean Tajima's D values for the autosomes (−0.742 ± 0.514 pre-treatment and −0.756 ± 0.513 post-treatment) as well as chromosome X (−0.880 ± 0.816 pre-treatment and −0.872 ± 0.816 post-treatment) were also similar between the two treatment groups (*p*-value = 0.8; one-sample Kolmogorov-Smirnov test) and no large-scale differences between the two treatment group pools were observed for chromosome 5 (Fig. 1e and f).

### 3.3 Window-based measures of genetic differentiation in response to ivermectin treatment

**Fig. 2:**
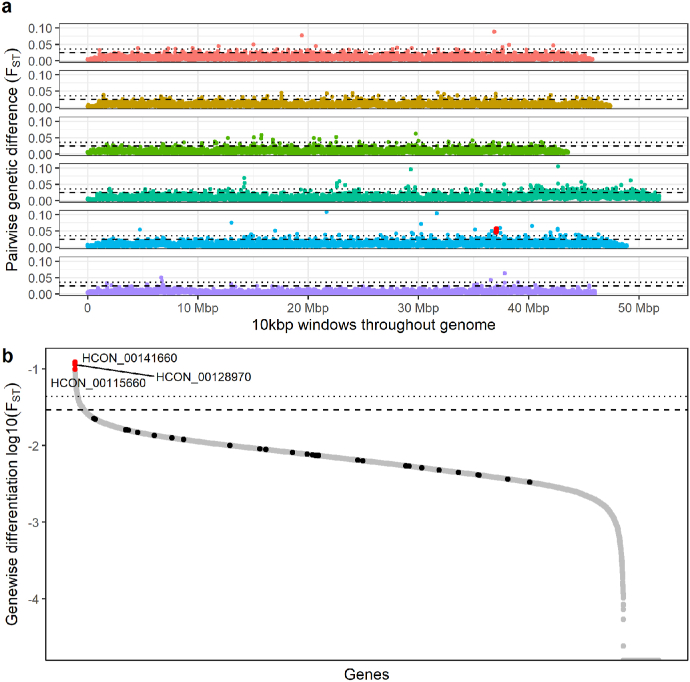
**Genetic differentiation between pre- and post-treatment groups.** Pairwise genetic differentiation between the two treatment groups was calculated as F_ST_ values per (a) 10 kbp windows throughout the genome or (b) for entire genes. In both (a) and (b), the level of significance is indicated by dashed black line (mean F_ST_ + 3 SDs) and dotted black line (mean F_ST_ + 5 SDs). (a) Three consecutive 10kbp windows above the mean F_ST_ + 5 SDs present in chromosome 5 are displayed in red. (b) F_ST_ values (left to right; in a decreasing manner) for genes *lgc-37, haf-6, osm-3, osm-5, lgc-55, pgp-9.1, pgp-9, avr-15, glc-1, avr-14, che-11, dyf-11, pgp-1, che-2, osm-1, lgc-36, mrp-1, che-3, pgp-12, che-12, glc-3, glc-2, che-13, glc-5, ggr-3, osm-6, pgp-3, unc-9, unc-38, dyf-7* are shown as black dots (standalone figure with gene names is shown as Supplementary Fig. 3), whereas the points in red represents the three top most F_ST_ value having genes (located in chromosomes 5 - HCON_00141660 and 4 - HCON_00128970 and HCON_00115660). Colours in (a) represent different chromosomes as indicated in the legend of Fig. 1. (For interpretation of the references to colour in this figure legend, the reader is referred to the Web version of this article.)

To identify treatment-induced genetic changes throughout the genome, pairwise genetic differences (F_ST_) between the two treatment group pools were estimated in 10 kbp windows throughout the genome, as well as for every single gene. Pairwise F_ST_ analysis revealed a low degree of differentiation between the two treatment groups (mean genome-wide F_ST_ = 0.0083; Fig. 2a; Supplementary Table 1). A total of 770 and 152 outlier 10 kbp windows were identified above 3 (F_st_ > 0.024) and 5 (F_ST_ > 0.035) SD from the genome-wide mean, respectively, most of which were present in chromosome 4 (n = 306 or n = 51, respectively). Of those 770 outlier windows, instances of at least 3 consecutive 10 kbp windows were recorded in 41 cases (26 of which were in chromosome 4), whereas only one such instance of at least 3 consecutive 10 kbp windows was observed above the mean F_ST_ + 5 SD - in chromosome 5, starting at 37.4 Mbp. Analyses of differentiation per gene also displayed a low degree of differentiation between the groups (mean F_ST_ = 0.007; Supplementary Table 2). We analyzed all genes in the genome to provide context for the analysis of F_ST_ from previously described candidate genes thought to be associated with IVM resistance (taken from Doyle et al., 2019); all candidates were within 3 SD of the genome-wide mean, of which the highest degree of differentiation was for *lgc-37* (F_ST_ = 0.022; Supplementary Table 3) (Supplementary Fig. 3). A total of 636 genes had an F_ST_ value at least as great as *lgc-37*, and a total of 92 genes showed substantial differentiation (F_ST_ > 5 SDs from genome-wide mean, i.e. F_ST_ > 0.043), of which the highest differentiation was observed for genes *HCON_00141660* (chromosome 5, ∼15 Mbp; F_ST_ = 0.122; Tajima's D_pre_ −1.17, Tajima's D_post_ −1.15), HCON_00128970 (chromosome 4, ∼48 Mbp; F_ST_ = 0.113; Tajima's D_pre_ −0.4, Tajima's D_post_ −0.5) and HCON_00115660 (chromosome 4, ∼26 Mbp; F_ST_ = 0.098; Tajima's D_pre_ −0.41, Tajima's D_post_ −0.4).

**Fig. 3:**
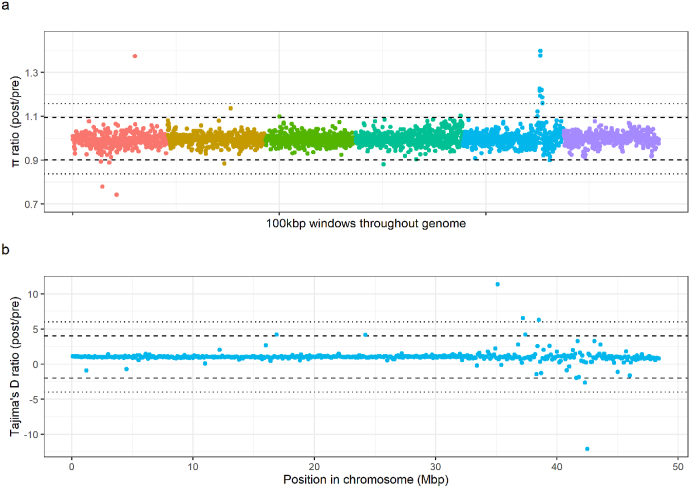
**Ratios of nucleotide diversity and Tajima's D highlight outlier variation in response to treatment in chromosome 5.** Genome-wide nucleotide diversity (a) and chromosome 5 Tajima's D (b) ratios were analyzed per every genomic 100 kbp window to identify subtle signs of selection in the post-treatment group. In both (a) and (b), the level of significance is indicated by dashed black line (mean ± 3 SDs) and dotted black line (mean ± 5 SDs). Colours represent different chromosomes as indicated in the legend of Fig. 1. (For interpretation of the references to colour in this figure legend, the reader is referred to the Web version of this article.)

In order to observe any subtle differences between the treatment groups, ratio comparisons per genomic 100 kbp windows between the ℼ values (i.e. post-treatment/pre-treatment) as well as Tajima's D were made. ℼ ratio comparison revealed a sudden increase in nucleotide diversity in the post-treatment group (up to roughly 1.35 × ) between 36.2 and 38.7 Mbp (mean ℼ value ratio + 3SDs; cut-off = 1.09; 217 overlapping genes) or 37.2–38.7 Mbp (mean ℼ value ratio + 5 SDs; cut-off = 1.15; 124 overlapping genes) in chromosome 5 (Fig. 3a). Further exploration of Tajima's D value ratio per every 100 kbp window in chromosome 5 showed that the differentiation of the values between the groups was observed to be highest at the terminal end of the chromosome (roughly 35–45 Mbp; 810 overlapping genes) wherein four outlier values (above mean Tajima D ratio + 5 SDs or below mean Tajima D ratio - 5 SDs) were found (Fig. 3b).

### 3.4 Analysis of single nucleotide variants in response to ivermectin treatment

We further analyzed allele frequency changes for every SNP by performing Fisher's exact tests (FET) on the pooled technical replicates (ie. pre-vs post) and Cochran–Mantel–Haenszel (CMH) tests on pairs of technical replicates (ie. 4 × pre-vs post) (Supplementary Figs. 1a and 1b). FET yielded roughly 2.7 million SNPs in the merged pre- and post-treatment groups, whereas the CMH test included 1.3 million SNPs throughout the replicate sample comparisons. Comparing the SNPs shared between the two tests (n = ～1.3 million; Supplementary Fig. 1c) revealed a high level of concordance between FET and CMH (Pearson's correlation; r = 0.97), but none of the changes in frequency were significant for both tests (*p*-value < 0.05; Bonferroni correction). A single SNP (in the non-coding part of the genome) in chromosome 4 (44,255,647 bp) showed significant changes in frequency above the genome-wide correction in the FET, but not in the CMH test.

*Bcftools* mediated SNP calling yielded roughly 1.3 million SNPs (roughly 1 variant every 213 bp), most of which were located in the non-coding regions of the genome (94%). In addition, around 73% of all SNPs occurring in the coding regions of the genome were synonymous. 436 out of the 770 (56%) and 60 out of the 152 (39%) outlier 10 kbp windows from the previous genetic differentiation analysis (in Fig. 2a) were found to harbor at least one non-synonymous mutation in the coding region of the genome, the majority of which (170 above mean + 3SD and 24 above mean + 5 SD; 39%) were clustered around the terminal region (40–50 Mbp) in chromosome 4 (Supplementary Fig. 2a). Outlier frequency changes for SNPs (with values above 3 or 5 SD of the genome-wide P-value mean) retrieved from the previous FET and CMH tests and resulting in missense mutations in the coding regions of the genome showed a cluster of these outlier values in chromosome 4 (29 values in CMH, i.e. 30% or 49 in FET, i.e. 28% > genome-wide mean + 5SD in the 40–51 Mbp region), again displaying an accumulation of non-synonymous SNPs in the terminal region of this chromosome (Supplementary Fig. 2). In contrast, frequency changes for non-synonymous SNPs present in the coding regions were few (5 in CMH test - 5%/9 in FET in region - 5% > genome-wide mean + 5SD) in the nucleotide diversity rich chromosome 5 terminal region (between 35 and 45 Mbp).

## 4 Discussion

In order to understand IVM resistance in *H. contortus*, relevant regions in the genome (QTL) as well as the underlying genetic changes within them which are responsible for the development of the resistant phenotype need to be defined. Here, we assessed the impact of IVM treatment on *H. contortus* from a Swedish farm population by sampling infective stage larvae (L3), recovered from the same animals, before and after the most recent IVM treatment and characterizing changes in genetic diversity genome-wide by pooled whole-genome sequencing. A key finding of this analysis was that a region around 36.2–38.7 Mbp on chromosome 5, consistent with a QTL previously implicated in IVM resistance (Doyle et al., 2019), was associated with IVM resistance in a new, geographically distinct population. Here we discuss our rationale, experiment and subsequent analyses in order to build upon the methodology used and make progress towards increasing our understanding of the mechanisms underpinning the development of IVM resistance.

A sheep farm on which reduced IVM efficacy had been previously suspected and the dominant species was determined to be *H. contortus* (≥90% of all recovered eggs) was selected for this study. We confirmed the resistance of the population by the FECRT (89% reduction estimate) and, therefore, hypothesized that a substantial change in the worm population after IVM treatment would correspond to large changes in the allele frequencies between the pre- and post-treatment populations, particularly in regions of the genome associated with resistance. Surprisingly, little genome-wide differentiation between the two groups was observed. Both pre- and post-treatment L3 pools revealed very similar genome-wide profiles for nucleotide diversity and Tajima's D estimates (Fig. 1a and b). On average, the estimates of Tajima's D (measured in 100 kbp windows throughout the genome) were below 0 in both treatment groups, suggesting that the population has undergone selective sweep(s) in the past. This is arguably not surprising, given that the field population had undergone multiple previous anthelmintic treatments in the recent past. Nevertheless, little is known about the unique evolutionary history of this population, making it difficult to understand the patterns of nucleotide diversity as well as Tajima's D existing prior to the current treatment with IVM. Consistent with loss of diversity due to recent sweeps, we identified relatively low levels of nucleotide diversity, which was approximately 10-fold lower in both treatment groups in comparison to other field-derived but laboratory-maintained strains, MHco4[WRS] and MHco10[CAVR] (Doyle et al., 2019). Although a relatively comparable raw read coverage was sequenced (120–137 × unmapped vs. 199.65 × raw read coverage in Doyle et al., 2019), a lower proportion of reads were mapped to the genome overall, which may be a consequence of either: (i) an inefficiency of mapping divergent sequencing data to the reference, especially around variable sites, (ii) a higher number of duplicated sequencing reads, reducing the effective mapped coverage; and/or (iii) a higher degree of homozygosity and/or reduced genetic variation in the studied population. The comparable levels of nucleotide diversity throughout the genome meant that measures of pairwise genetic differentiation by F_ST_ revealed little deviation between the two groups; although minor sporadic differentiation throughout the genome was observed (i.e., we did identify 152 10 kbp windows greater than 5 SDs from the genome-wide mean), the population pre- and post-treatment was broadly genetically similar. The maintenance of genetic diversity in the post-treatment group suggests that resistant alleles are likely to be present on multiple, different genetic backgrounds in the studied field population, which would reflect that the population itself has gradually evolved to be resistant over time with sufficient admixture between resistant and susceptible worms, rather than from a recently acquired *de novo* mutation which has spread quickly in the population. In addition, since the post-treatment samples were collected 7 days after ivermectin exposure, the residual effects of the drug could have affected egg deposition rather than worm survival. This in turn could help explain the unexpected genetic similarities between the two treatment groups. Nevertheless, studies seem to indicate only a short-term (up to 3–5 days) inhibition of nematode egg production upon ivermectin treatment (Scott et al., 1991; McKenna, 1997; Sutherland et al., 1999), effectively suggesting that temporary egg deposition suppression after treatment is unlikely to be of much significance here. Despite the genetic similarities between the groups, we attempted to find peaks of differentiation (F_ST_) across the six chromosomes, which we defined as at least three consecutive 10 kbp windows above mean F_ST_ + 3 SD/5 SD. Whilst we observed multiple (n = 41) minor peaks (>mean F_ST_ + 3 SD; 26 present in chromosome 4), only a single major peak (>mean F_ST_ + 5 SD) was found in chromosome 5 beginning at around 37.4 Mbp (Fig. 2a; red dots in the chromosome 5 part of the panel), suggesting that the highest degree of consistent, genome-wide genetic differentiation between the groups is present within the previously suggested region containing the major QTL of IVM resistance (Doyle et al., 2019).

Many genes have been proposed to be associated with ivermectin resistance. However, high levels of genetic diversity as a consequence of large effective population sizes and the high fecundity of *H. contortus*, particularly in field populations, complicates the genetic association between resistance phenotype and discovery of causal mutations. This is particularly evident in recent genome-wide analyses of anthelmintic resistance (Doyle et al., 2019; Niciura et al., 2019; Sallé et al., 2019; Khan et al., 2020), and is consistent with the data presented here. An analysis of genetic differentiation among the top proposed candidates between pre- and post-treatment did not show any of these genes to be among the statistical outliers (>3 SD from the genome-wide mean). Our data suggests 636 genes with higher genetic differentiation than the most differentiated among the candidates, *lgc-37*, leading us to question the role (if any) of *lgc-37* and resistance here. The three highest F_ST_ estimates were obtained for genes *HCON_00141660* (orthologue to *vap-1* in *C. elegans*)*, HCON_00128970* and *HCON_00115660*, the latter two of which had no orthologues in other nematode species (according to WormBase Parasite) in addition to being located in chromosome 4 (48943064–48943459 bp; F_ST_ = 0.113 and 26890603–26892649 bp; F_ST_ = 0.098); while there was not convincing evidence of a broader selection footprint around these regions in either the genome-wide differentiation (F_ST_) or nucleotide diversity analyses, a single SNP (pos: 44,255,647 bp) in the non-coding region of the genome and close to the location of *HCON_00128970* in chromosome 4 was statistically significant in the FET (but not CMH) analysis. In addition, having investigated the outlier 10 kbp windows (wherein F_ST_ value > genome-wide mean F_ST_ + 3 or 5SD) and outlier SNP frequency changes (wherein P-value > genome-wide mean P-value + 3 or 5SD in FET and CMH test) for the presence of overlapping non-synonymous SNPs in the coding region of the genome, we found clusters of non-synonymous variants at the terminal region of chromosome 4 (Supplementary Fig. 2; around 40–51 Mbp). These data suggest that the terminal end of chromosome 4 and perhaps *HCON_00128970*, even though its function or relation to resistance is not known, require further investigation.

Pairwise comparison of nucleotide diversity did, however, identify a single outlier region around the 36.2–38.7 Mbp region (mean ℼ value ratio + 3 SDs) in chromosome 5 which contained a 1.35 × increase in diversity in the post-relative to pre-treatment group. While the direction of this change was surprising, i.e. the post-treatment pool was more diverse than the pre-treatment, we hypothesize that this pattern may be associated with the improved detection of low-frequency variants after removal of a large proportion of the population after treatment within a region which had previously undergone a selective sweep. Our hypothesis is based on the fact that this region coincides with the major IVM QTL previously reported in chromosome 5, identified via genetic backcrossing of resistant alleles into a susceptible genetic background (Doyle et al., 2019). Comparison of the Tajima's D values between the two groups showed no substantial pattern of change. Nevertheless, the most extreme variation (window values above/below mean ± 5 SDs) in the ratio of Tajima's D was observed in the downstream terminal region of chromosome 5 (around 35–45 Mbp), suggesting that this region is somewhat more genetically diverse or undergoing some degree of change (Fig. 3b). If our hypothesis is correct, it would further support our hypothesis that resistant alleles are on diverse genetic backgrounds, and that they have been present in this population of parasites for some time.

While significant progress is being made toward mapping drug resistant-associated variation using genetic crosses (Choi et al., 2017; Doyle et al., 2019, 2021; Niciura et al., 2019), the validation of these data in field populations is critically important towards the development of molecular markers of resistance (Kotze et al., 2020). However, the analysis of variation in the field, even from a genome-wide perspective, still presents significant challenges as we have demonstrated here. Although our approach - to sequence pools of larvae pre- and post-treatment from a single farm - was simple in theory, there was still substantial biological and technical variation within the experiment resulting in increased variation within the sequencing data. Unlike experiments involving genetic crosses where genetic variation for a particular trait segregates in a controlled environment, studies of field populations are more heavily influenced by the larger effective population sizes and variation in the underlying genetic structure of parasite populations. *H. contortus* is highly genetically variable (Sallé et al., 2019) and, therefore, it can be reasonably expected that some changes between populations, even between two different time points from the same population (in the case of this study), can arise due to reasons unrelated to resistance (Gilleard and Beech, 2007). Although we expected to account for this variation to some degree by studying a single population, recovered as paired samples from the same animals before and seven days after IVM treatment, our approach could be improved by using time-matched but drug-naive controls (i.e. sampling an untreated group at the same time as the treated group, before and after treatment), as well as studying larvae recovered from individual hosts (as opposed to larvae pooled across hosts, as was performed here) in order to account for genetic differences not involved in resistance (i.e. time and host effects). Although a clear reduction in the egg counts was observed upon performing the FECRT (89% average reduction), the obtained value is only indicative of the efficacy of the treatment and, therefore, it would be sensible to employ *in vitro* tests (such as egg hatch or larvae development tests) to determine if the surviving fraction is truly resistant to the effects of the drug. In addition, another viable approach would be to sample multiple farms with different response phenotypes and correlate the genetic and phenotypic responses. In this way, stochastic noise (i.e. genetic variability arising due to reasons other than resistance) could be addressed, thereby reducing the frequency of false positive signals and amplifying the real signal behind resistance. Finally, while we were able to identify a moderate level of differentiation specifically in a previously identified IVM QTL, the overall genetic similarity may also reflect selection occurring at multiple independent genetic loci and that the experimental approach together with a pool-sequencing based analysis is underpowered to detect very small shifts in genetic variation at multiple loci. While increasing evidence suggests this is not the case (Doyle et al., 2019, 2021), we expect that significant variation in field populations would likely provide the necessary variation upon which selection can act to overcome the negative effects of drug exposure and may do so through multiple mechanisms. We note that we have only examined genomic and not transcriptomic variation in our data, which may provide additional insight into the direct and/or indirect consequences of drug exposure and subsequent development of resistance (Laing et al., 2021). As technologies become cheaper and more accessible, analysis of populations of individual phenotypically well-defined parasites using whole-genome and/or transcriptome sequencing will help to resolve the question of the contribution of major and minor loci toward resistance.

Collectively, the data obtained here suggests that, despite a significant reduction in egg counts post-treatment, the genetic diversity of a single field population of *H. contortus* sampled before and after treatment is maintained throughout the genome. Importantly, we do find evidence of genetic differentiation in chromosome 5, which lends further support for the previously identified IVM QTL (Doyle et al., 2019) in a new genetically and geographically distinct population of *H. contortus.* While we have not identified the causal variant, the validation of this QTL in a new field population continues to refine our understanding of IVM resistance, as well as identify a number of key areas of focus and improvement which future research should consider for mapping resistance alleles in the field.

## Declaration of competing interest

The authors of this manuscript certify that they have NO affiliations with or involvement in any organization or entity with any financial interest, or non-financial interest in the subject matter discussed in this manuscript.

## Appendix A Supplementary data

Multimedia component 4 Multimedia component 4

figs1 figs1

figs2 figs2

figs3 figs3
