# Supplementary material for: Genome-wide analysis of the response to ivermectin treatment by a Swedish field population of Haemonchus contortus
Source: Int J Parasitol Drugs Drug Resist. 2021 Dec 23;18:12–9. doi: 10.1016/j.ijpddr.2021.12.002 (PMC8718930; doi:10.1016/j.ijpddr.2021.12.002)
Supplement: Multimedia component 1 [file mmc1.docx]

**Supplementary figure 1.** Fisher’s exact test (a) and CMH test (b) were employed to estimate any significant, SNP frequency changes (-log_10_[*p*-value]) between the two treatment groups. Colors represent different chromosomes as indicated in the legend of figure 1. (c) -log10(P) values for common SNPs obtained through performing FET and CMH tests were plotted together (on separate axes) in order to identify significant allele frequency changes of variants in both tests. Points in black represent SNPs occurring only in the coding regions and resulting in non-synonymous mutations, whereas points in gray represent all other SNPs (i.e. either synonymous, in the coding region occurring, or in the non-coding region occurring SNPs). The dark red lines in (above each subplot in all three panels) represent genome-wide Bonferroni’s correction, calculated for 95% significance level.

**Supplementary figure 2.** Annotated, non-synonymous SNPs (in the coding regions of the genome) found overlapping with outlier (> mean F_ST_ + 3 or 5 SD) 10 kbp windows in the pairwise genetic differentiation analysis (F_ST_; Figure 2a) as well as obtained as outliers (> mean P-value + 3 or 5 SD) in the previous FET (b) and CMH (c) tests. (a) Dots in dark gray and black represent outlier windows above the genome-wide mean F_ST_ + 3 and +5 SDs (respectively) and wherein at least one non-synonymous mutation in the coding region is found. (b) and (c) Data points (SNP frequency changes) appearing in gray represent values from Supplementary figure 1 above the mean P-value + 3 SD, whereas SNP frequencies in black are above the mean P-value + 5 SD. 1-6 on the side of each subplot refer to chromosomes 1 through 5 and X.

**Supplementary figure 3.** Standalone figure for figure 2b. Gene-wise genetic differentiation between the two treatment groups was calculated as F_ST_ values per entire genes (gray dots). F_ST_ values for various, previously thought candidate genes for IVM resistance (*lgc-37, haf-6, osm-3, osm-5, lgc-55, pgp-9.1, pgp-9, avr-15, glc-1, avr-14, che-11, dyf-11, pgp-1, che-2, osm-1, lgc-36, mrp-1, che-3, pgp-12, che-12, glc-3, glc-2, che-13, glc-5, ggr-3, osm-6, pgp-3, unc-9, unc-38, dyf-7*) are shown as black dots, whereas the points in red represents the three top most F_ST_ value having genes (located in chromosomes 5 - HCON_00141660 and 4 - HCON_00128970 and HCON_00115660 ). The level of significance is indicated by dashed black line (mean F_ST_ + 3 SDs) and dotted black line (mean F_ST_ + 5 SDs).

**Supplementary table 1.** Genome-wide genetic differentiation (F_ST_) between the pre- and post-treatment pools, estimated in 10kbp sliding windows throughout the genome. The three columns correspond to a chromosome number (1), step size in a window (2) and F_ST_ value for each (3).

**Supplementary table 2.** Gene-wise (i.e. per gene) differentiation (F_ST_) throughout the genome between the pre- and post-treatment pools. Column 1 corresponds to the official gene name and column 2 – Fst-value for that gene.

**Supplementary table 3.** Gene-wise (i.e. per gene) differentiation (F_ST_) for previously proposed candidate genes, between the pre- and post-treatment pools. Column 1 corresponds to the candidate acronym, column 2 – to the official candidate gene name and column 3 – F_ST_ value for each gene.
