# Supplementary figures and images for: Genome-wide analysis of the response to ivermectin treatment by a Swedish field population of Haemonchus contortus
Source: Int J Parasitol Drugs Drug Resist. 2021 Dec 23;18:12–9. doi: 10.1016/j.ijpddr.2021.12.002 (PMC8718930; doi:10.1016/j.ijpddr.2021.12.002)

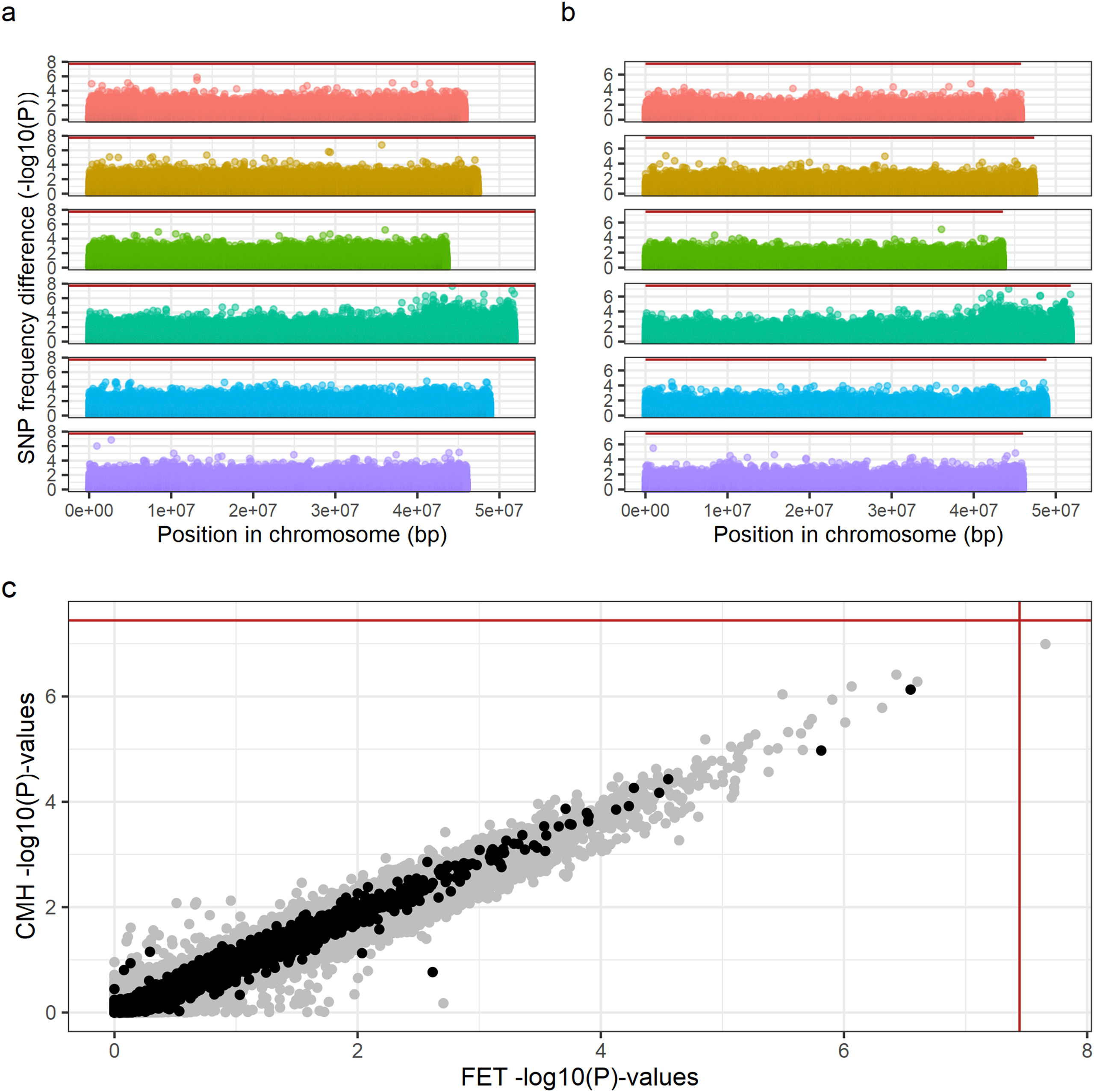

Supplement: figs1 [file mmcfigs1.jpg]

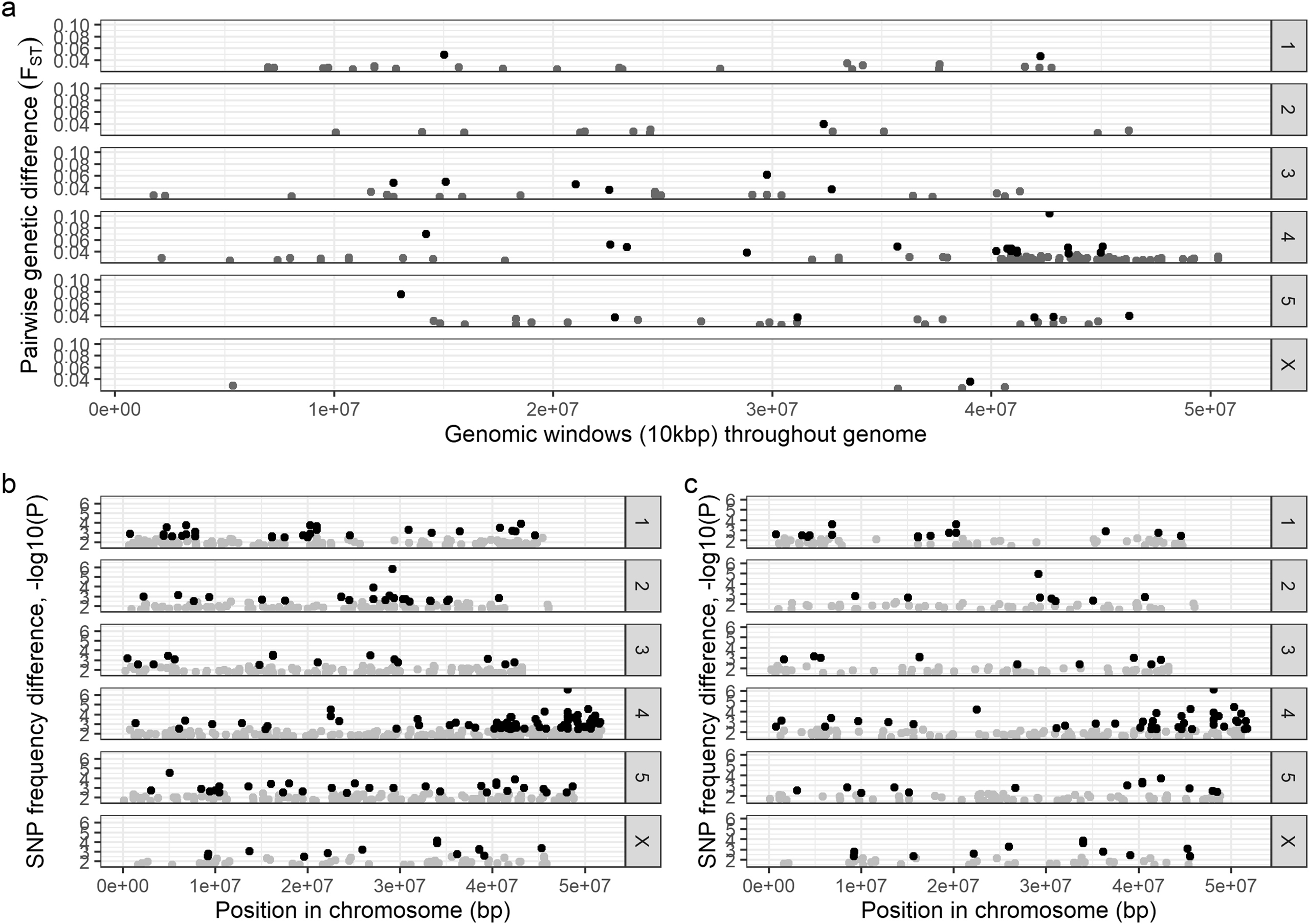

Supplement: figs2 [file mmcfigs2.jpg]

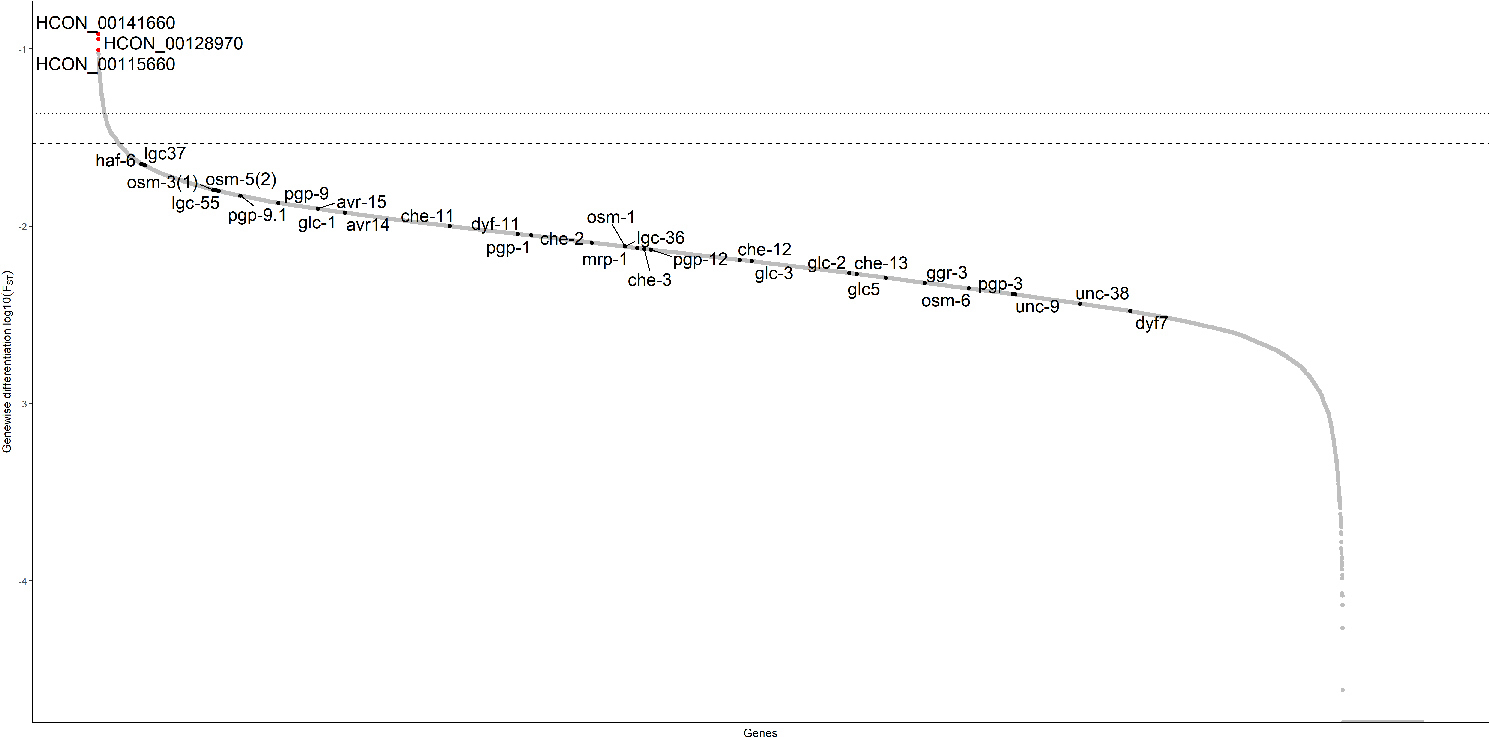

Supplement: figs3 [file mmcfigs3.jpg]
